# STARDUST: a pipeline for the unbiased analysis of astrocyte regional calcium dynamics

**DOI:** 10.1101/2024.04.04.588196

**Published:** 2024-04-08

**Authors:** Yifan Wu, Yanchao Dai, Katheryn B. Lefton, Timothy E. Holy, Thomas Papouin

**Affiliations:** 1 Washington University in St. Louis, Department of Neuroscience, St. Louis, MO 63110 USA; 2 Technical contact; 3 Lead contact

## Abstract

Calcium imaging has become an increasingly popular way to probe the activity of astrocytes. However, the governing principles of astrocyte calcium dynamics are still elusive and their relationship to cellular events ill-defined. Useful assumptions and ‘shortcuts’ commonly applied to neuronal recordings therefore do not hold true for astrocytes. The imaging of astrocyte calcium activity per se can be relatively straightforward, subsequent analysis methods that adequately capture the richness and complexity of calcium dynamics remain scant. Here, we introduce STARDUST, a pipeline and python-based data processing for the Spatio-Temporal Analysis of Regional Dynamics & Unbiased Sorting of Transients from astrocyte calcium recordings. STARDUST builds upon AQuA to identify patches of active signals, from which it builds a data-driven map of regions of activity (ROAs) that can be combined with cell-segmentation and/or correlated to cellular morphology. For each ROA, STARDUST extracts fluorescence time-series, and performs signal identification and features extraction. STARDUST is agnostic to cell morphology (or cells altogether) and putative calcium propagation across ROAs. Instead, it focuses on decomposing calcium dynamics in a regionalized fashion by treating ROAs as independent units, for instance allowing investigations by signal feature-based ROA rank. STARDUST also identifies ROAs as “stable” (active throughout), “ON” (turned on during drug application) and “OFF” (turned off during drug application) in pharmacology experiments, permitting studies of astrocyte calcium “micro-domains” based on their functional responses. With a systematic set of instructions and troubleshooting tips, and minimal computational/coding background required, STARDUST is a user-friendly addition to the growing toolbox for the exploration of astrocyte calcium dynamics.

## Before you begin

### Overview

Calcium imaging allows the optical probing of neural activity. It relies on the detection of changes in intracellular calcium concentration ([Ca^2+^]_i_), by means of fluorescent calcium indicators, and while it has been predominantly deployed in neurons, it is applicable to a host of cells that populate the nervous system. Among them, astrocytes, a main type of non-neuronal cells in the brain, display spontaneous [Ca^2+^]_i_ fluctuations that are responsive to ambient conditions and neuroactive molecules.^1–3^ Like in neurons, this form of signaling has thus been used as a mean to assess astrocytes activity and measure their responsiveness to a variety of conditions within neural circuits, in vitro, ex vivo and in vivo.^1–3^ However, important differences between neurons and astrocytes mean that proven methods of neuronal calcium imaging and analysis are not transferable to astrocytes.

In neurons, calcium activity is driven by depolarization-induced transmembrane calcium fluxes, which allows straightforward interpretations of Ca^2+^-indicator data.^4^ Typically, Ca^2+^ions enter the soma during action potential firing, through voltage-gated calcium channels, making somatic calcium imaging an excellent proxy of neuronal firing. Alternatively, Ca^2+^ ions flowing through other channels (e.g., as NMDA receptors) provide a localized read-out of membrane depolarization in specific compartments (e.g., dendritic spines). By contrast, the mechanisms underlying astrocyte [Ca^2+^]_i_ variations are diverse and still largely elusive.^1–3^ This strongly limits our ability to tie calcium signals to particular cellular events, or assume ground-truths about their rules of propagation and integration. This means that calcium imaging remains a loosely defined proxy of astrocyte activity.^5^ Additionally, the soma of neurons is a useful hub for sampling neuronal activity and the influence of external inputs because of the propagative nature of membrane depolarization events and their summation at the axonal initial segment. Astrocytes, however, lack such properties. In fact, astrocytic [Ca^2+^]_i_ fluctuations occur primarily outside of the soma, within highly localized micro/nano-domains, and seldom propagate or merge at the cell body^6–9^, such that imaging somatic calcium is not a suitable way to capture astrocyte dynamics or ‘excitability’. However, the complexity and sub-diffraction limit scale of astrocyte morphology also means that it is virtually impossible to make morpho-functional annotations in live 2-photon laser scanning microscopy (2-PLSM) imaging of astrocytes i.e., to register areas of [Ca^2+^]_i_ activity to identifiable morphological features akin to spines. Considering how popular astrocyte calcium imaging has become, there is thus a need for new or adapted methods and models to analyze calcium activity in such recordings. Owing to the constrains enumerated above, such methods need to be 1) comprehensive, to capture all activity in the cell, 2) unbiased, rather than restricted to user-defined regions of interest, 3) agnostic, to make no or minimal assumptions regarding the spatio-temporal properties of underlying Ca^2+^ fluxes, and 4) with instrument-limited accuracy, to favor detailed investigations consistent with the structured nature of astrocytes architecture^10^.

Here, we introduce STARDUST, a pipeline and python-based algorithm for the Spatio-Temporal Analysis of Regional Dynamics and Unbiased Sorting of Transients from astrocyte calcium image stacks. STARDUST performs motion correction and minimal preprocessing, and builds upon AQuA, a popular open-source fluorescence analysis platform^11^, to identify patches of active signals from which it builds a map of regions of activity (ROAs). We show how the map of ROAs can be combined with further cell-segmentation and/or correlated to cellular morphology, even though this is optional for STARDUST output. STARDUST can yield as many as 100 or more ROAs per cell (depending on imaging conditions), or thousands across the field of view, and extract fluorescence time-series, signals and signal features from each of them. Importantly, STARDUST makes no assumption regarding putative calcium propagation across ROAs, in line with the seemingly static nature of astrocyte Ca^2+^ activity^6–9^, but in contrast with AQuA where signals that “coherently occur in a spatially connected region” are lumped together to provide an event-based readout^11^. Instead, STARDUST treats ROAs as independent units to focus on decomposing calcium dynamics in a regionalized fashion, allowing ROA-based as well as cell-based analysis of transient properties. It allows the ranking or categorizing of ROAs based on the properties of the transients occurring within them, and the detailed analysis of subsequent manipulations depending on such ranks. A particularly useful instantiation of this feature is in pharmacology experiments, where STADUST can separate “stable ROAs” (that are active throughout the recording), from “ON ROAs” (that are inactive at baseline and turn on during drug application) and “OFF ROAs” (that are active at baseline and turn off during drug application). Together, this makes STARDUST a user-friendly complement or alternative to the small number of publicly available algorithms and tools recently developed^6,8,11–13^ to tackle astrocyte calcium dynamics. This protocol provides a step-by-step guide on how to use STARDUST, a systematic walkthrough of its core functionalities, limitation, and tunability, and outlines multiple examples of results it can yield.

### Hardware

A Microsoft Windows machine with 32GB is recommended. AQuA does not currently support Mac OS and Linux system.

### Software

#### Download AQuA

AQuA is a software package originally designed to decompose raw dynamic astrocyte imaging data into a set of quantifiable ‘events’.^11^ Here, however, AQuA is only used to extract pixel with above-threshold fluorescence independent of any region of interest or underlying cell morphology, a feature for which it performs very well.

1. Download AQuA from https://github.com/yu-lab-vt/AquA. Follow the instruction in the section “Download and installation” to download the “MATLAB GUI” version to a local directory.
2. Extract all files from the zip folder “AQuA-master”.
3. To learn more about AQuA, a step-by-step user guide is available in the section “Getting started” on the GitHub page.

#### Download MATLAB to run AQuA

- Download MATLAB from https://www.mathworks.com/products/matlab.html. Critical: MATLAB 2017a or later is required to run AQuA.
- Install MABLAB. During installation, install the following toolboxes required for AQuA: image processing toolbox, statistics and machine learning toolbox, and curve fitting toolbox. Alternatively, install these toolboxes in “Add-Ons” in the “HOME” tab inside MATLAB.

#### Download ImageJ/Fiji for image processing

- Download Fiji from https://fiji.sc/.

#### Download Anaconda python environment

The STARDUST custom-made code is written with Python. Anaconda is a python environment that consists of an interpreter and any number of installed packages. Using Anaconda saves effort and time in installing python basic packages.

- Download Anaconda from https://www.anaconda.com/download.

#### Download Python and Visual Studio Code (VS code)

This protocol uses VS code for code editing, highlighting, and debugging. Other script editors such as Jupiter Notebook, Spider also work with the python code.

- Download the latest version of Python from https://www.python.org/downloads/.
- Download the VS code from https://code.visualstudio.com/docs/setup/windows and the python extension from https://marketplace.visualstudio.com/items?itemName=ms-python.python. Check “Auto search for environment” in the installation prompt.
- Follow the instruction for using Python in VS code here https://code.visualstudio.com/docs/python/python-tutorial, look for the section that uses Anaconda as the environment. Additionally, here is another instruction for selecting the correct environment https://code.visualstudio.com/docs/python/environments.

### Data collection

One channel image stack in TIFF format is supported by AQuA. If the raw image file is in different format, open the raw image file in ImageJ and convert it to TIFF by *Save as > Tiff file* in ImageJ.

## Institutional permissions

All experiments were conducted in accordance with the guideline of the Institutional Animal Use Committee of Washington University in St. Louis School of Medicine (IACUC #20180184 and 21-0372, Animal Welfare Assurance #D16-00245).

## Step-by-step method details

This section describes the step-by-step process for 1) image preprocessing (including motion correction and noise filtering), 2) map of region-of-activity (ROA) generation, 3) fluorescence time series extraction, 4) cell mask segmentation and cell mask acquisition, and finally 5) signal detection and feature extraction with custom-made python-based analysis pipeline.

To better illustrate each step, we use two-photon laser scanning microscopy (2-PLSM) recordings of the calcium sensor lck-GCaMP6f expressed in astrocytes (AAV5-gfaABC1D:lck-GCaMP6f, micro-injected at P70) in hippocampal acute slices obtained from adult mice at P100.

### Image Preprocessing

This step includes movie quality check and motion correction. Because astrocyte Ca^2+^ activity occurs predominantly in small nano/micro-domains^6–9^ that are both confined and difficult to tie to any anatomically defined sub-cellular structure (except for signals occurring in primary branches and soma), astrocyte Ca^2+^ recordings and subsequent analysis do not tolerate spatial drift. We thus strongly recommend applying a motion correction against noticeable drift in the recording. If permitted, a second-channel recording of a static cell marker (e.g. GFAP-mCherry reporter) can be used as a reference for image registration.

1. Open the TIFF image file in ImageJ.
2. Play the image stack and check the following:
  - Signal to noise ratio
    - Open *B&C window* from *Image>Adjust>Brightness and Contrast.* Click “Auto” or adjust the slide bars to tune the contrast. A good recording should have clear and bright signals that stand out from the background noise.
  - Shift during recording and registration
    - Identify an anatomical feature, such as a cell body or a blood vessel, as a visual reference to check x,y,z drift. If there is significant drift over time, i.e. the x,y location of the signals shift away from their original footprint, the video will require a registration. In case of a z-shift outside of the original focal plan (i.e. out of focus), the video might not be suitable for analysis (see below). Tip: compare the last frame and first frame to assess the extent and direction of the drift.
    - TurboReg is a great ImageJ plugin for motion correction. It can be downloaded from https://bigwww.epfl.ch/thevenaz/turboreg/. However, due to the highly diverse nature of calcium activity in astrocytes, the “Bulk processing” function of TurboReg occasionally fails to achieve an adequate registration. Therefore, the registered recording should be carefully examined before proceeding to next step.
    - Since this protocol focuses on single-plane recordings, if there is significant shift along the z-axis, we suggest removing the recording from analysis. A significant z-shift is one where most or all of the initial hotspots and anatomical features (soma, branches, blood vessel) are lost from the field of view (compare first and last frame).
3. Check the recording to assess overall fluorescence change over time, such as increased or decreased baseline/background activity or fluorescence bleaching over time: *Image> Stacks > Plot Z-axis Profile*. This is also particularly useful when pharmacological treatments are performed, to narrow down the time of drug application.

### Active pixels detection

This section uses AQuA, a publicly available software package, to identify active pixels that are above noise threshold. All supra-threshold active pixels will be color-labeled after analysis.

1. Launch MATLAB. In the “Current Folder” toolbar, navigate to the folder where the file “AQuA-master” is saved. Add the “AQuA-master” folder to the working directory by right clicking the folder name in the “current folder” window, select “Add to Path” and “Selected Folders and Subfolders”.
2. Launch AQuA by entering “aqua_gui” in the Command window. A popup window of AQuA with “New project” and “Load existing” will appear. Click “New project” and a field for file selection and parameter options will show as in Fig. 2. Input the image file to analyze by clicking the three dots on the right.
3. Preset analysis parameters are determined according to the Data type selected. In the “Data type” drop-down menu, select the type of imaging condition in the corresponding data file. In our example image, the data is collected from acute brain slice with GCaMP6f-Lck calcium indicator, and we choose “GCaMPExVivoLck”. After selecting the data type, pre-selected values will be automatically filled for the three relevant parameters at the bottom. Edit the temporal resolution and spatial resolution if needed according to recording conditions.

**Critical:** For “Exclude pixels shorter than this distance to border”, change to “**0**” to ensure that the output file has the exact same canvas size as the raw file. This step is critical for accurately matching up the map of ROA back to the same spatial footprints in the raw file.

- Click “Open” to proceed to the analysis window.
- The top left box “Direction, regions, landmarks” includes functions such as defining cell boundary or regions or creating anatomical mask. This step can be applied to exclude unnecessary regions or restrict the analysis to regions of interest. Removing unnecessary pixels reduces processing time in AQuA. Here we provide an example of how to select a specific region for analysis. In this example, we are interested in analyzing the region outlined in yellow (Fig. 3) and would like to exclude other regions in the analysis. Here is how to do so in AQuA:
  - Select “Mask builder”, then in the pop-up window, select “Self” under the “Region” (Fig. 4). “Self” means the selected video will be used as a reference to build the mask. After clicking “Self”, some regions are automatically selected if the pixels are above a default intensity threshold. To build a new mask that outlines the region of interest, clear the default region by clicking “Clear” and then click “Add” to draw the region of interest by hand under the “Manually Select” section. And then click “Apply & back”. If successfully selected, the movie in the AQuA main window will have the region(s) outlined and numbered.
  - Please refer to the AQuA user guide for other ways to determine desired regions in the field of view for analysis.
- Select steps for AQuA analysis. In the detection window on the left, check the box “skip step 2,3,4” to detect active pixel without further processing and merging. This step is crucial because the STARDUST pipeline analyzes raw active pixel, while the full-length AQuA platform assigns voxel that coherently occur in a spatially connected regions into “events” based on their spatio-temporal properties and assumptions that are not necessary in STARDUST.
- Set the parameters for active pixels detections. There are three parameters: intensity threshold scaling factor, smoothing and minimum size. Default numbers set by the selected data type in the step 6 provide a good starting point for parameter selections. However, to prevent signal under-sampling (omitting true signals) or over-sampling (taking in noise), it is crucial to optimize parameters based on movie properties, such as signal to noise ratio. For example, the intensity scaling factor can be fine-tuned by comparing the true signals in the input video with the color-labeled signals in the output. Increase the scaling factor if excessive noise is detected and decrease it if true signals are omitted. Minimum size can be determined by finding the smallest pixel groups that give meaningful signals, i.e. that exhibit real signals, not noise, in the raw images. Based on examples presented later in this protocol, we recommend an intensity threshold scaling factor of 2.5, smoothing (sigma) 0.5, and minimum size 10 for processing.
- Click “Run all steps” to run the pipeline. A proof-reading window and export window will appear once the software finishes processing.
- Set the filter condition for the output file. This step is crucial for ROA mask optimization. Select the parameter “Area”, which is the size of the active zones in μm^2^, as the filter criteria by checking the adjacent box in the “Proof reading” window (Fig. 5A). The range of area needs to be optimized to capture as many ROAs and signals as possible. We strongly recommend benchmarking different ranges of area min and max cutoffs because the optimal range will depend greatly on the imaging conditions and image stack quality. Filter criterion that is too stringent will lead to the loss of active regions, while criteria that is too loose will combine/merge adjacent regions into a single ROA leading to a loss of spatial resolution and a decreased of signal to noise ratio of the dF/F time-series as illustrated in Fig. 5. We recommend starting with an area range of 10 μm^2^ to 40 μm^2^. Export the movie with color-labeled active signals overlay. To obtain the output movie with only color-labeled signals, in “Movie brightness/contrast” window, set the brightness of the raw video to zero, and in the “Feature overlay” window, set the brightness of the overlay image to max.
- Save the output file. In the export window on the left, uncheck the “Events” and “Feature table” box, leaving only the “Movie with overlay” box checked. Click “Export/Save” to save the file in the targeted folder.

### Region-of-activity (ROA) map acquisition

This section generates the map of regions of activity (ROAs) from the AQuA output movie. We define an ROA as the individual aggregate spatial footprint of active pixel patches in the temporal projection of the image stacks. As a result, 1) an ROA is an area within which at least one active pixel patch occurs during a recording, 2) active pixel patches do not need to occur over the entire footprint of the ROA to which they belong, 3) the footprints of two active pixel patches from a same ROA do not necessarily overlap, as it is the aggregate footprint of all active pixel patches that defines the boundaries of the ROA, 4) any two given ROAs are spatially unconnected, 5) the constellation of ROAs for a cell represents an unbiased map of spatially independent zones of activity within that cell. Additionally, 6) the activity of two contiguous ROAs is assumed to be independent, except for activity that might co-occur if the ROAs fall within the spatial footprint of an active pixel patch that has been excluded due to the maximum ROA area cutoff criteria. The advantages of this analysis method are that it is unbiased, semi-automated, comprehensive, and solely activity-based in that it is decoupled from (but compatible with) morphological constrains or cell-based criteria.

1. From here, we switch to ImageJ for further processing. Acquire the map of ROA by performing a t(z) projection on the output file from AQUA. Open the file in ImageJ. Click *Image>Stacks>Z Project>Max intensity* to generate a t-projected image.
2. Assign each ROA as a region of interest for analysis in ImageJ. Convert the t-projected ROA map to binary image by *Process>Binary> Make Binary*. Then select *Analyze>Analyze particles*, set the size to 0-infinity. Also check the boxes of “Include holes”, “Add to manager” and “Composite ROIs”. Click “OK”.
3. In the ROI manager, each ROA is assigned a number and highlighted with yellow boundary in the image. The total number of the ROAs can be found as the last number in the ROI manager.
4. Generate a binary ROA mask for later pipeline processing. Select all the ROIs in the ROI manager (Ctl+A), right click and select “OR (Combine)”, then “Add” to the ROI manager. This generates an ROI that combines all the ROI selections. Select this ROI and then go to *Edit > Selection> Create Mask*. A binary mask with all of ROAs shown as white area is generated. Save this mask with the name ROA_mask under the same file folder by clicking File > Save as> Tiff file.
5. Save all the ROI selections. Select all ROIs and right click to save as RoiSet.zip. This is helpful if changes need to be made later.

### Time-series data acquisition

This section extracts time-series fluorescence intensity from each ROA in the ROA map.

1. Open the original raw signal file in Tiff form. Overlay ROI selection onto the original file by clicking “Show All” in the ROI manager.
2. Set measurement item in ImageJ > Analyze >Set measurements. Check the box “mean gray value”. This measures the intensity of the selected ROIs.

**Note**: The area of each ROA can be obtained with the same method by checking the box “Area” instead. This is of interest when comparing ROA features (numbers, size, etc) across different conditions (e.g., control vs KO).

- Select all the ROI selections in the ROI manager, right click and select “multi measure”. In the next popup window, select “measure all slices” and “one row per slice”. A result window will then appear with the values of intensity for each ROA over time.
- Copy and paste the data from the result window into an excel file. Save as .csv file format.

### Cell mask acquisition

This step identifies the cell boundaries in the field of view to obtain the list of cells to be considered for analysis. Note that this step is optional and is not required to process downstream analysis.

1. Obtain a reference image for cell boundary in the recording field of view. Because of the complex structure of astrocytes, determining the cell boundary for each cell is intrinsically challenging. We offer two suggestions to obtain a reference image.
  - If multichannel recordings or images from a second, static marker was acquired: the reference image can be obtained from them.
  - If no static marker was used: a reference image can be obtained by generating a Z-projection from the original t-stack recording by Image>Stacks>Z projection. Because baseline fluorescence in the lck-GCAMP is usually low, and because cyto-GCaMP primarily reveals astrocytes soma and branches, the arborization of individual astrocytes may not appear in full in either case. However, this might suffice to outline the general shape of astrocytes. To improve cell segmentation, we strongly recommend using sparse expression of GCaMP (usually by reducing viral titer or tamoxifen dosage).
2. Delineate cell boundary with the “Polygon selections” tool. Overlay the ROIs selections onto the reference image and include each ROA in their respective cell. Add each cell selection to the ROI manager by “Ctl +t” or “Add” in the ROI manager. Because astrocyte domains have virtually no or minimal spatial overlap, cell boundaries should not touch each other. Ideally, all ROAs should be ‘assigned’ to a cell (i.e., included within a cell boundary), since astrocytes tile the entire neuropil. However, by virtue of their 3D tiling, it can be challenging to determine whether a set of processes/ROAs belongs to an adjacent astrocyte within the focal plan or to an astrocyte outside of the focal plan.
3. Generate the cell mask. Select all the cell selections in the ROI manager, right click and select “OR (Combine)”, then “Add” to the ROI manager. This generates the ROI that includes all the cell selections. Select this ROI and then go to *Edit > Selection>Create Mask*. Save it as Cell_mask.tiff file under the same file folder.
4. Save all the ROI selections. Select all ROIs and right click to save as RoiSet.zip.

Notes:

1. To this point, ROA_mask.tiff, Cell_mask.tiff and time-series data.csv files have all been generated. These are the three input files for downstream signal detection and analysis with the STARDUST python pipeline.
2. The feature of this protocol is to analyze the regional activity of astrocytes. If the analysis focuses on the response from the entire cell (for instance in the case of the response to norepinephrine), the steps that consist in obtaining the active pixels and creating map of ROAs may be skipped. Instead, the analysis should be as follow: 1) Image preprocessing, 2) Cell mask acquisition step 1–3, 3) Time-series data acquisition from cell selection. The required input file for signal detection is the time-series data.csv file.

### Signal detection with customized code

This section introduces the following modules in the customized python code: signal preprocessing, baseline determination, signal detection, pipeline check points, ROA grouping and data output. Notes on how to optimize key parameters are also highlighted. Practical instructions on code execution are annotated for each section inside the code.

### Signal preprocessing

This step provides optional signal smoothening and detrending. However, due to the enigmatic and dynamic nature of astrocyte calcium activity, signal preprocessing may alter or eliminate true features of calcium activity and therefore should be used with moderation, great caution, and adequate rationale.

1. To smoothen fluorescence signals, we choose a 4^th^ order low-pass Butterworth filter with a cutoff frequency of 0.4 Hz and apply this filter to the signal traces using “filtfilt”, which filters the signal in a forward and backward direction to remove phase distortion. “butter” and “filtfilt” are functions from the “scipy.signal” module in Python. The low-pass filter is set to remove the high-frequency noise, while the cutoff frequency is set to ensure a minimal modification of the traces (Fig. 6).
2. To correct potential baseline shifts that result from micro-movement or minor drifts in the recording, a linear trend is subtracted from the original trace to obtain a corrected trace (Fig. 7). Linear regression is preformed using the module “stats.linregress”. The degree of correction can be adjusted from 0 to 1 which represents the percentage of linear trend to be subtracted in the function.

**Note**: Linear regression is best used to remove a trend shared by all time-series traces from a given recording (which is an indication that an overall drift occurred during the recording). By contrast, we advise that an upward or downward drift in only a sub-selection of ROAs might be indicative of a local or cell-specific change in baseline calcium levels, which is potentially biologically relevant and therefore should not be corrected. Additionally, beware that large transients tend to bias the linear regression calculation. Lastly, there exists other sophisticated methods for signal correction. Please select the optimal methods in specific conditions.

### Baseline determination

This part of the code determines the true baseline of each trace, which is a prerequisite for accurate signal detection and features extraction. We use an iterative method to refine the baseline value that consists in repeating a three-step process: 1) baseline calculation 2) signal segments identification 3) signal segments removal. As shown in Fig. 8 this rapidly converges onto the true baseline.

1. Baseline calculation. For the first iteration, a default baseline is calculated by averaging the entire trace. This initial baseline calculation yields a value that can be far from the true baseline but will allow the identification of the largest transients in the time-series. In the second iteration and onward, baseline is calculated as the mean of signal-free traces.
2. Signal segments identification. By applying a threshold determination (baseline + 2SD), a first selection of trace segments that contain signals is detected. We call these “signal segments”.
3. Signal segments removal. The signal segments detected in step 2 are omitted for next round of baseline calculation.

By running steps 1 to 3 iteratively, the baseline rapidly converges on a value corresponding to the mean F value of an entire trace devoid of signals exceeding the signal detection threshold criteria. We find that convergence occurs within 5 iterations with no further improvement (and no deviation) if additional iterations are done (Fig. 8).

### Signal detection

This part of the code executes functions that permit setting a signal detection threshold, identifying signal segments, and extracting signal features, such as peak amplitude, area under the curve (AUC), duration, rise time, decay time, and frequency.

**Figure 1.**
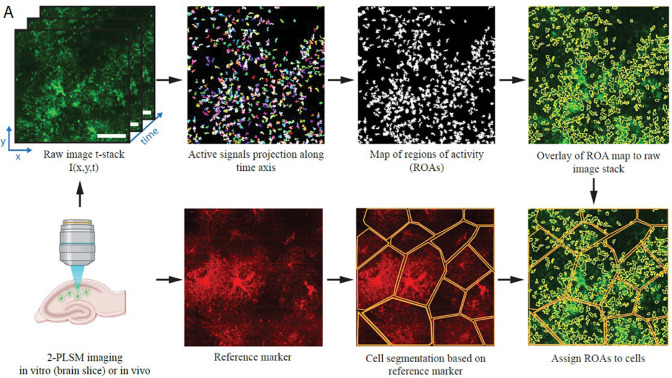
STARDUST workflow. Illustration of the steps of the STARDUST pipeline described in this protocol. Note that cell segmentation (bottom) is optional.

**Figure 2.**
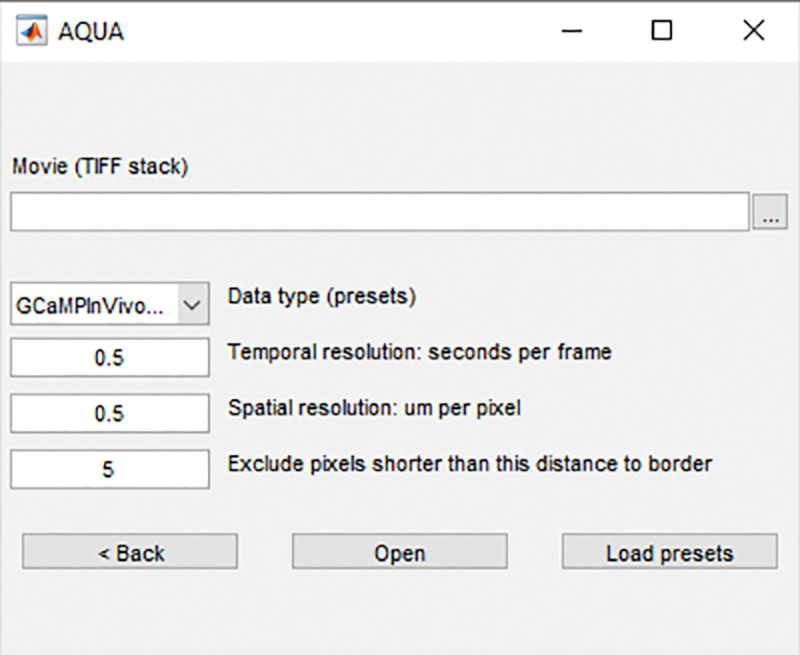
The AQuA launching window.

**Figure 3.**
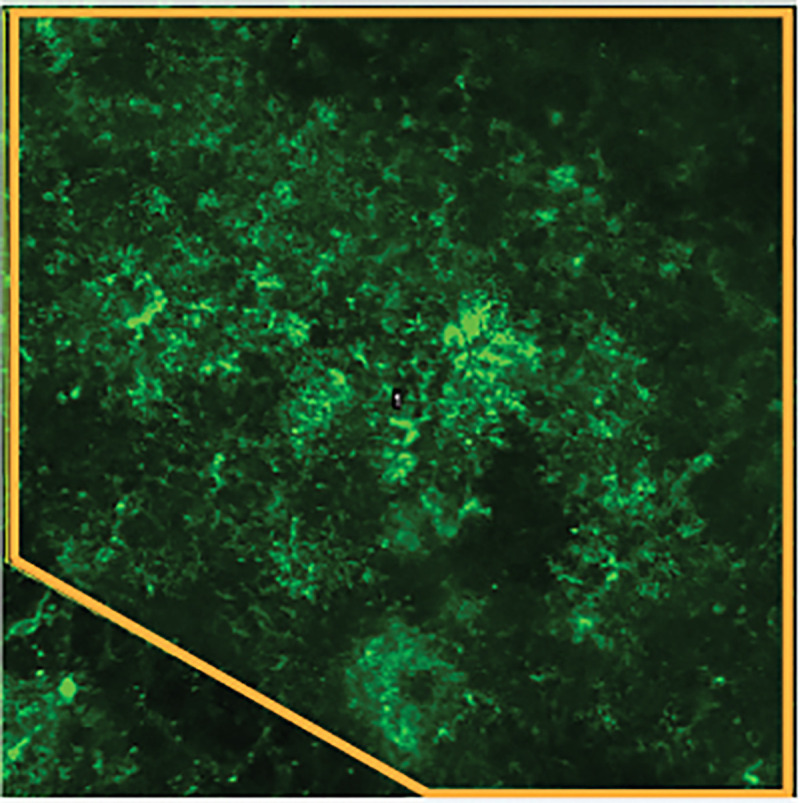
A region of interest for subsequent analysis is drawn (orange) on the image stack reference.

**Figure 4.**
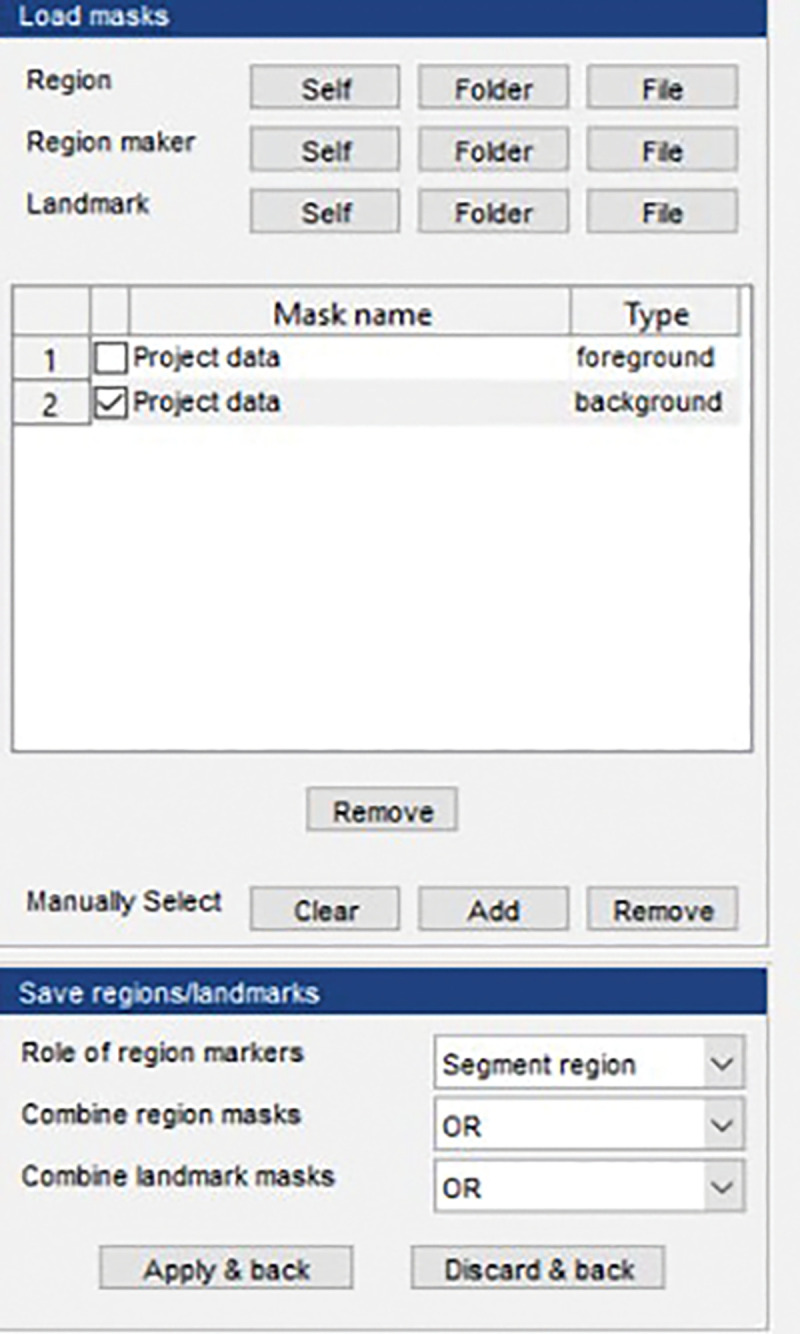
The mask builder interface.

**Figure 5.**
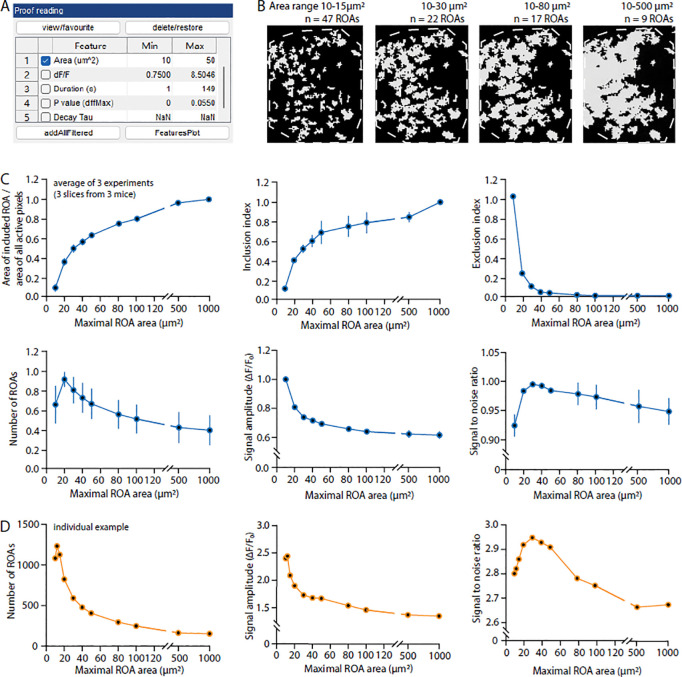
Determining the adequate range of ROA sizes. A, “Proof reading” window in AQuA for selecting appropriate filter criteria of the output movie. B, Effect of the min and max cutoffs values of the “Area” parameter on the map of active zones (yellow areas) for a representative cell (dotted outline). The number of ROAs eventually obtained with each settings is indicated. Note how, as active zones get larger, they tend to fuse. Ultimately, this results in large but fewer ROAs. C, Quantification of the effect of the max Area cutoff setting on diverse parameters. The inclusion index specifically measures how ROAs, for a given maximal area criteria, successfully capture larger active pixel patches that are filtered out by this setting. It is measured as the percentage of the spatial footprint (pixels) of excluded larger pixel patches captured by the final map of ROAs. The exclusion index measures, for each setting, the number of active pixel patches (if no cutoff is applied) that have zero pixels included in the final map of ROAs. D, Individual examples of the effect of maximum area cutoff on the final number of ROAs, average signal amplitude and signal to noise ratio. Note the drastic effect at the level of individual experiments. Peak or optima may occur at very different max area values across experiments, which tends to minimize the effect of the max area setting when averaging across experiments in (C).

**Figure 6.**
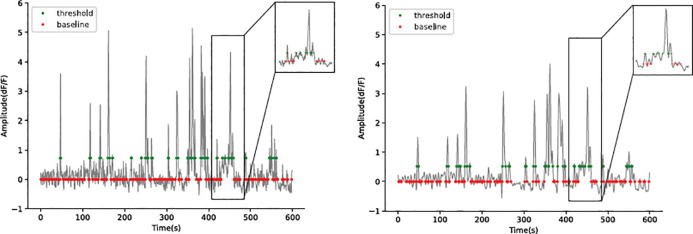
Filtering, or not filtering. *Left*: raw signal; *Right*: signal after Butterworth filtering. Note the marginal improvement in signal detection while the amplitude of transients is moderately diminished. Due to noise reduction, however, the 2SD threshold (see below) is significantly lowered, which directly influences signal detection. In this example, we would argue against filtering. Red dots indicate when the trace crosses the baseline. Green dots indicate when the trace crosses the signal detection threshold.

1. Once the baseline is determined, a normalized trace “ΔF/F_0_ trace” can be calculated as: <mi>.
2. Choose an adequate threshold criterion for signal detection based on your data (such as signal/noise ratio or other biologically guided criteria). We, and others, base this criterion on the standard deviation (SD) calculated from the signal segments devoid of signals. In this case, we use 2SD. To be more conservative, use 3SD.
3. Once the threshold is set, a self-defined “find_roots” function is applied to identify the “baseline crossing points”, when the trace intersects the average baseline (red dots in Fig. 6 and Fig. 1), and the “threshold crossing points”, when the trace intersects signal threshold (green dots in Fig. 6 and Fig. 1). The bounds of a signal correspond to the closest “baseline crossing points” that flank the corresponding “threshold crossing points”.

**Note**: Per this design, signals that lack an initiation or termination bound will not be captured for analysis (Fig. 9). This might result in some ROAs having no signals, even though they were derived from an active pixel patch.

- Extract signal features. Peak amplitude (dF/F) is determined by using a self-defined “findpeaks” function which finds the maximal point within a signal segment. The area under the curve (AUC, dF/F·s) is determined using the “integrate.simpson” function which calculates the integration area under the trace. Halfwidth (S) is the time between the two 50% peak amplitude crossing points. Duration is determined as the time elapsed between the signal initiation and signal termination (baseline crossing points). The rise time is determined as the time elapsed between the 10% peak and 90% peak crossing points. The decay time is determined as the time elapsed between the 90% peak and 10% peak crossing points. Please note that these definitions depart slightly (but importantly) from those typically employed for neuronal recordings, wherein the decay time is often the time constant of the exponential fit applied to the “90%–10% of peak” portion of the trace. However, few astrocyte Ca2+ signals appear to display EPSC- or EPSP-like kinetics, and in most Ca2+ recordings of astrocyte activity the sampling rate (~1Hz) is often too low for an adequate exponential regression.

### Data visualization and checkpoints

This section introduces several checkpoints in the code that allow users to visualize their data and refine parameters as needed prior to proceeding farther.

**Figure 7.**
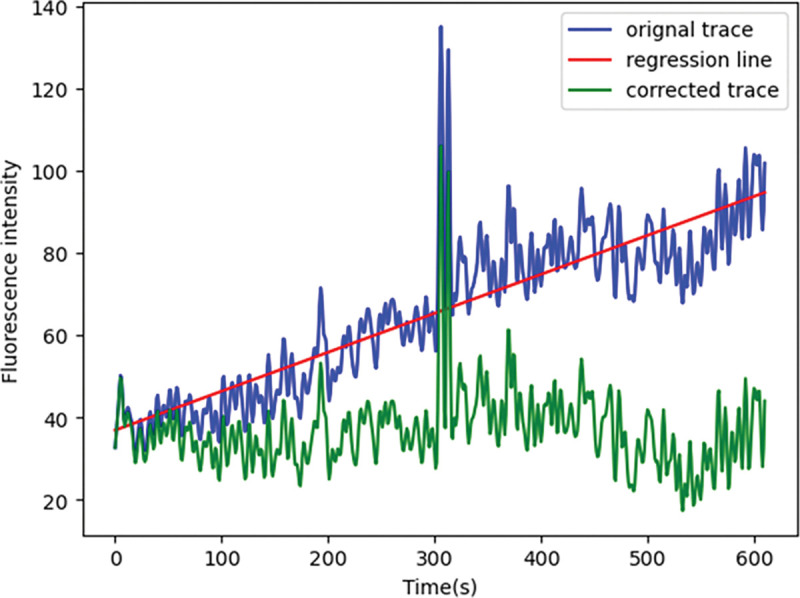
The upward shift in the original trace (blue) is corrected by subtracting a linear trend (red line). The corrected trace (green) is used to subsequent analysis.

**Figure 8.**
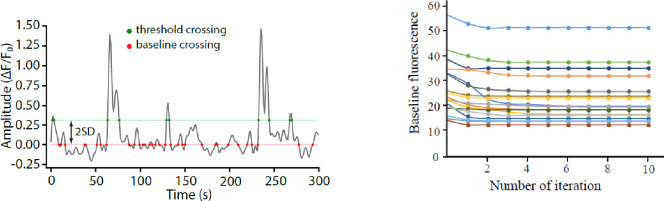
Baseline determination. *Left*, Illustration of a fluorescence time series extracted from a single ROA, showing baseline crossing points (red line and red dots) and signals detection threshold crossing points (green line and dots). 2SD: 2 standard deviations. *Right*, The baseline value systematically converges after less than 5 iterations (usually 3). Each trace shows the average baseline fluorescence (F_0_) of 17 individual time series traces over 10 cycles of baseline determination.

**Figure 9.**
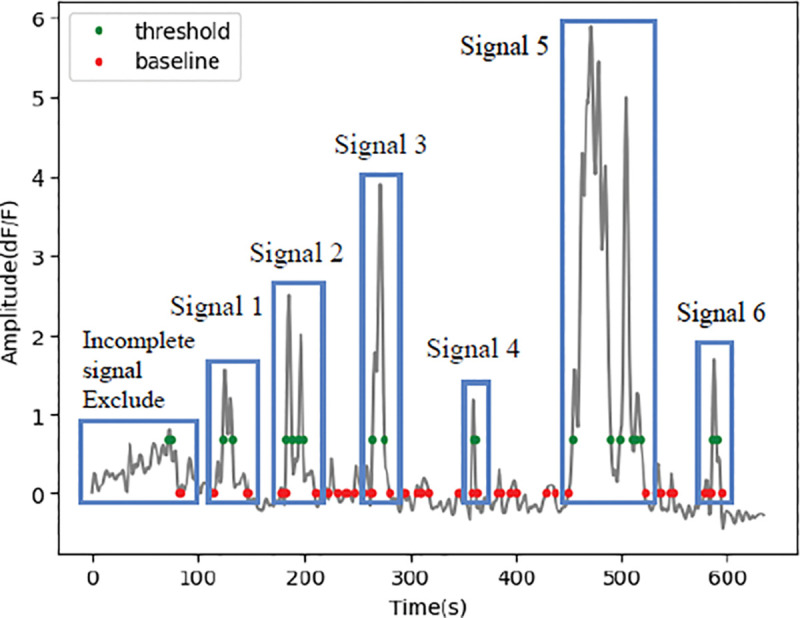
Signal extraction. Signal bounds are determined by the nearest baseline crossing points flanking a threshold crossing point. Incomplete signals without an initiation point or a termination point are excluded. In this example trace, 6 signals are included for analysis, and one is excluded.

1. The first checkpoint is located at the trace correction section (Signal preprocessing - Step 2). It allows users to visually inspect raw data traces. If signal correction is applied (Signal preprocessing - Step 2), a plot illustrated in Fig. 7 is displayed with the raw trace, regression line and corrected line.
2. The second checkpoint is at the end of the Signal detection section, where the trace is shown as in Fig. 6 and Fig. 9. Users can inspect the baseline determination, number of signals included etc.

**Note**: The run time is usually <60 seconds per 500 ROAs processed. Enabling the data visualization step may increase the run time. A convenient way to screen traces without increasing runtime is to run the code in an interactive window in the VS code.

### Assigning ROAs to cells

This step reads in the two binary masks-ROA_mask.tiff and Cell_mask.tiff -and assigns each ROA to the cell that shares the same spatial footprint. This step is applied to obtain a cell-based analysis by grouping the ROAs of a given cell together.

1. The two images are first converted to NumPy arrays.
2. The connected pixels in the two binary images are labeled with an unique integer using the “label” function from the “scipy.ndimage”. By doing so, the pixels of interest in the two masks are highlighted.
3. A dictionary named “region_map” is initialized to store the mapping between ROAs and cells.
4. Iterate over each ROA region and find the corresponding large regions in the cell mask. The result is stored in the “region_map” as of “ROA_number: Cell_number”, e.g.ROA_1: Cell_2.
5. Generate a dataframe from the dictionary “region_map” to analyze ROA features such as number of total ROAs, number of cells, and number of ROAs in respective cell.

### Data output

The results of the analysis are all compiled as “pandas dataframes” and exported as an Excel file with multiple datasheets. Depending on the analysis results, these datasheets include, but are not limited to, a master sheet with all features analyzed for each signal, an extensive summary for ROA-based analysis, a summary of cell-based analysis calculated from ROAs within respective cell boundary, a full list of ΔF/F_0_ traces for all ROAs, and a full list of cell-based ΔF/F_0_ traces averaged from ROAs within the cells for all cells.

**Note**: Important parameters, such as the time of drug application, can also be included in this section for further analysis.

### Expected outcomes

**Figure 10.**
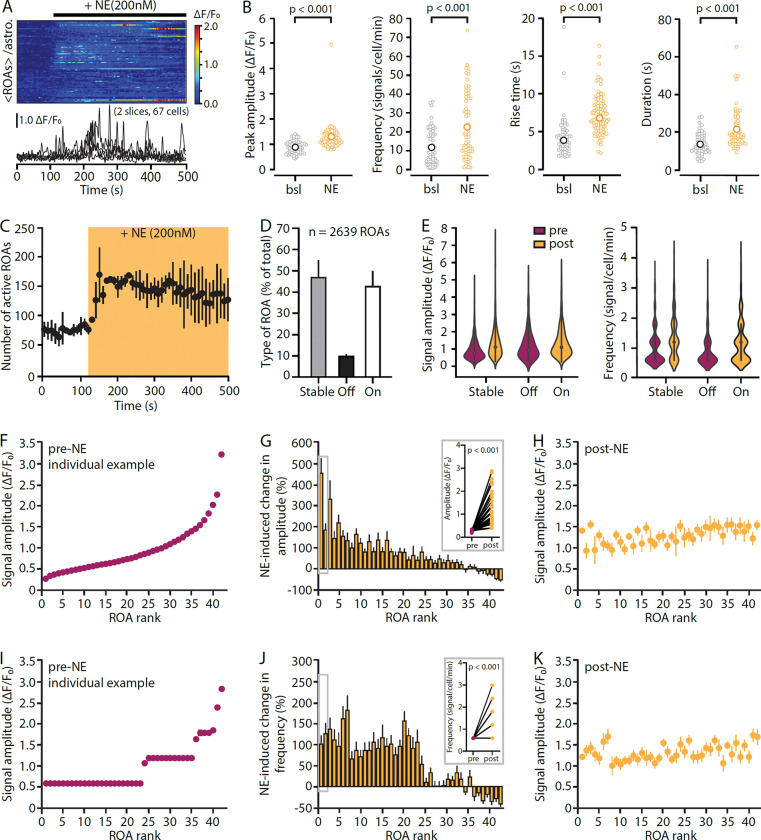
Example data from cell-based and ROA-based analysis of calcium responses to norepinephrine (NE, 200nM). **A**, Kymograph (individual rows show averaged ROA fluorescence per astrocyte) and dF/F_0_ traces (± s.e.m.) of spontaneous astrocyte Ca2+ activity in before and during NE application. **B**, Pots of the amplitude, frequency and kinetics of Ca2+ transients during baseline (bsl) and NE application. Data shown as averaged of all ROAs per cell. Permutation tests were used. **C**, Effect of NE application on the number of active ROAs overtime in hippocampal brain slices (AAV5-gfaABC1D::lck-GCaMP6f transduced astrocytes) from adult mice (P100). (continues on next page) **legend, continued**. **D**, Proportions of “Stable”, “On”, and “Off” ROAs that are, respectively, active solely prior to NE application, solely after NE application or during the course of the entire recording. Note the large proportion of ROAs specifically recruited by NE. **E**, Transients amplitude and frequency per ROA type. Amplitude: Stable-pre vs. post p<0.001, vs. Off p<0.001, vs. On p<0.001, Frequency: Stable-pre vs. post p<0.001, vs. Off p<0.05, vs. On p<0.001 (linear mixed effect models). **F-H**, Ranking ROAs based on their transients peak amplitude prior to NE application (F, 42 bins, 10 ROAs each) reveals that NE exerts a differential effect across ROA types (G). Specifically, the potentiating effect of NE on transients amplitude is more pronounced in low-rank ROAs, which displayed the smaller transients under control conditions, and largely absent in high-rank ROAs. As a result, transients amplitude is uniformed across ROA ranks in the presence of NE (H). Inset in G shows paired data for the first two ranks. Data are obtained from 100s of pre-NE condition (0–100s) and 100s of post-NE condition (200–300s). **I-K**, Same as F-H with transient frequency. H-J: Percent change in frequency per ROA rank (J) shows a clearer cutoff point.

In Fig. 10 below, we illustrate some of the data that STARDUST yields in a classic experimental paradigm. Specifically, acute hippocampal brain slices were obtained from adult mice (P100) in which astrocytes were transduced with an AAV5-gfaABC1S::lck-GCaMP6f at P70. GCaMP6f activity in stratum radiatum astrocytes was recorded under 2-PLSM (NIKON A1RHD25 MP microscope, 920nm excitation, 25X, 1.1 NA, 1.6x optical zoom) in response to the perfusion of low concentrations of norepinephrine (NE, 200nM). Panels A-B shows a classical cell-based analysis of transient properties in response to NE a pairwise fashion. Panels C-K provides examples of ROA-type and ROA-rank based analyses for the same experiments.

## Article info

### Resource availability

#### Lead contact

Further information and requests for resources and reagents should be directed to and will be fulfilled by the lead contact, Thomas Papouin (thomas.papouin@wustl.edu).

#### Technical contact

Questions about the technical specifics of performing the protocol should be directed to and will be answered by the technical contact, Yifan Wu (yifan.wu@wustl.edu).

#### Materials availability

This study did not generate new unique reagents.

#### Data and code availability

Original code will be available upon request.

## References

[1] Semyanov A., Henneberger C., and Agarwal A. (2020). Making sense of astrocytic calcium signals - from acquisition to interpretation. Nat Rev Neurosci 21, 551–564. 10.1038/s41583-020-0361-8. 32873937

[2] Shigetomi E., Patel S., and Khakh B.S. (2016). Probing the Complexities of Astrocyte Calcium Signaling. Trends Cell Biol 26, 300–312. 10.1016/j.tcb.2016.01.003. 26896246 PMC4946798

[3] Rusakov D.A. (2015). Disentangling calcium-driven astrocyte physiology. Nat Rev Neurosci 16, 226–233. 10.1038/nrn3878. 25757560

[4] Grienberger C., Giovannucci A., Zeiger W., and Portera-Cailliau C. (2022). Two-photon calcium imaging of neuronal activity. Nat Rev Methods Primers 2, 67. 10.1038/s43586-022-00147-1. 38124998 PMC10732251

[5] Murphy-Royal C., Ching S., and Papouin T. (2023). A conceptual framework for astrocyte function. Nat Neurosci 26, 1848–1856. 10.1038/s41593-023-01448-8. 37857773 PMC10990637

[6] Srinivasan R., Huang B.S., Venugopal S., Johnston A.D., Chai H., Zeng H., Golshani P., and Khakh B.S. (2015). Ca(2+) signaling in astrocytes from Ip3r2(−/−) mice in brain slices and during startle responses in vivo. Nat Neurosci 18, 708–717. 10.1038/nn.4001. 25894291 PMC4429056

[7] Arizono M., Inavalli V.V.G.K., Panatier A., Pfeiffer T., Angibaud J., Levet F., Ter Veer M.J.T., Stobart J., Bellocchio L., Mikoshiba K., (2020). Structural basis of astrocytic Ca2+ signals at tripartite synapses. Nat Commun 11, 1906. 10.1038/s41467-020-15648-4. 32312988 PMC7170846

[8] Agarwal A., Wu P.-H., Hughes E.G., Fukaya M., Tischfield M.A., Langseth A.J., Wirtz D., and Bergles D.E. (2017). Transient Opening of the Mitochondrial Permeability Transition Pore Induces Microdomain Calcium Transients in Astrocyte Processes. Neuron 93, 587–605.e7. 10.1016/j.neuron.2016.12.034. 28132831 PMC5308886

[9] Bindocci E., Savtchouk I., Liaudet N., Becker D., Carriero G., and Volterra A. (2017). Three-dimensional Ca2+ imaging advances understanding of astrocyte biology. Science 356, eaai8185. 10.1126/science.aai8185. 28522470

[10] Salmon C.K., Syed T.A., Kacerovsky J.B., Alivodej N., Schober A.L., Sloan T.F.W., Pratte M.T., Rosen M.P., Green M., Chirgwin-Dasgupta A., (2023). Organizing principles of astrocytic nanoarchitecture in the mouse cerebral cortex. Curr Biol 33, 957–972.e5. 10.1016/j.cub.2023.01.043. 36805126

[11] Wang Y., DelRosso N.V., Vaidyanathan T.V., Cahill M.K., Reitman M.E., Pittolo S., Mi X., Yu G., and Poskanzer K.E. (2019). Accurate quantification of astrocyte and neurotransmitter fluorescence dynamics for single-cell and population-level physiology. Nat Neurosci 22, 1936–1944. 10.1038/s41593-019-0492-2. 31570865 PMC6858541

[12] Bjørnstad D.M., Åbjørsbråten K.S., Hennestad E., Cunen C., Hermansen G.H., Bojarskaite L., Pettersen K.H., Vervaeke K., and Enger R. (2021). Begonia—A Two-Photon Imaging Analysis Pipeline for Astrocytic Ca2+ Signals. Front. Cell. Neurosci. 15. 10.3389/fncel.2021.681066. PMC8172593 34093134

[13] Dzyubenko E., Prazuch W., Pillath-Eilers M., Polanska J., and Hermann D.M. (2021). Analysing Intercellular Communication in Astrocytic Networks Using “Astral.” Front Cell Neurosci 15, 689268. 10.3389/fncel.2021.689268. 34211372 PMC8239356

